# Hypoxia-Inducible Lysine Methyltransferases: G9a and GLP Hypoxic Regulation, Non-histone Substrate Modification, and Pathological Relevance

**DOI:** 10.3389/fgene.2020.579636

**Published:** 2020-09-03

**Authors:** Anand Chopra, William C. Cho, William G. Willmore, Kyle K. Biggar

**Affiliations:** ^1^Institute of Biochemistry, Carleton University, Ottawa, ON, Canada; ^2^Department of Biology, Carleton University, Ottawa, ON, Canada; ^3^Department of Clinical Oncology, Queen Elizabeth Hospital, Hong Kong, China

**Keywords:** G9a, G9a-like protein, GLP, lysine methylation, non-histone, hypoxia

## Abstract

Oxygen sensing is inherent among most animal lifeforms and is critical for organism survival. Oxygen sensing mechanisms collectively trigger cellular and physiological responses that enable adaption to a reduction in ideal oxygen levels. The major mechanism by which oxygen-responsive changes in the transcriptome occur are mediated through the hypoxia-inducible factor (HIF) pathway. Upon reduced oxygen conditions, HIF activates hypoxia-responsive gene expression programs. However, under normal oxygen conditions, the activity of HIF is regularly suppressed by cellular oxygen sensors; prolyl-4 and asparaginyl hydroxylases. Recently, these oxygen sensors have also been found to suppress the function of two lysine methyltransferases, G9a and G9a-like protein (GLP). In this manner, the methyltransferase activity of G9a and GLP are hypoxia-inducible and thus present a new avenue of low-oxygen signaling. Furthermore, G9a and GLP elicit lysine methylation on a wide variety of non-histone proteins, many of which are known to be regulated by hypoxia. In this article we aim to review the effects of oxygen on G9a and GLP function, non-histone methylation events inflicted by these methyltransferases, and the clinical relevance of these enzymes in cancer.

## Introduction

In 2019, a trio of scientists was jointly awarded the Nobel Prize in Physiology or Medicine “*for their discoveries of how cells sense and adapt to oxygen availability*” (Nobel Media AB 2020, 2019). Collectively, Drs. William G. Kaelin Jr., Sir Peter J. Ratcliffe and Gregg L. Semenza pioneered early research efforts to shed light on this vital biological phenomenon. Indeed, deciphering of the underlying biochemical mechanisms have since led to our current canonical understanding of how oxygen-sensitive enzymes and cellular machinery coordinate together to “turn-off” the major regulatory protagonists at play; the hypoxia-inducible factor (HIF) proteins. In the 1990s, it was discovered that the HIF1 protein can be controlled by the availability of molecular oxygen (i.e., O_2_). This finding began the search for how oxygen mechanistically acts as a signal for HIF1 regulation (Semenza et al., 1991; Wang et al., 1995). The state of knowledge at the time was that low-oxygen intracellular signaling events were likely initiated through protein phosphorylation; a well-documented post-translational modification (PTM) of the era (Graves and Krebs, 1999; Ardito et al., 2017). Yet the mechanism triggering low-oxygen signaling was eventually pinpointed not to be through phosphorylation, but instead via protein hydroxylation, specifically prolyl hydroxylation (Maxwell et al., 1999; Ivan et al., 2001; Jaakkola et al., 2001). Though PTMs outside the realm of phosphorylation were lesser documented at the time, modifications involved in signal transduction are now known to extend to, but are not limited to, hydroxylation, acetylation, ubiquitination and methylation (Rahimi and Costello, 2015). While still in its infancy, the role of lysine methylation in signal transduction has been burgeoning since the beginning of the twenty-first century and is now of particular interest regarding its role in oxygen-responsive cellular signaling.

The emergence of lysine methylation as a signaling PTM has revealed novel complexities in the regulation of well-studied signal transduction pathways (Biggar and Li, 2015; Batista and Helguero, 2018; Levy, 2019). Importantly, lysine methylation is known to play critical regulatory roles in cancer-promoting pathways and methyl-specific regulatory enzymes present as druggable targets for therapeutic design (McGrath and Trojer, 2015; Carlson and Gozani, 2016). The methylation of lysine residues is dynamically controlled by enzymes that facilitate the addition and removal of methyl (-CH_3_) groups at the ε-amino group of lysine residues; lysine methyltransferases (KMTs) and lysine demethylases (KDMs), respectively (Paik et al., 2007; Smith and Denu, 2009).

**FIGURE 1:**
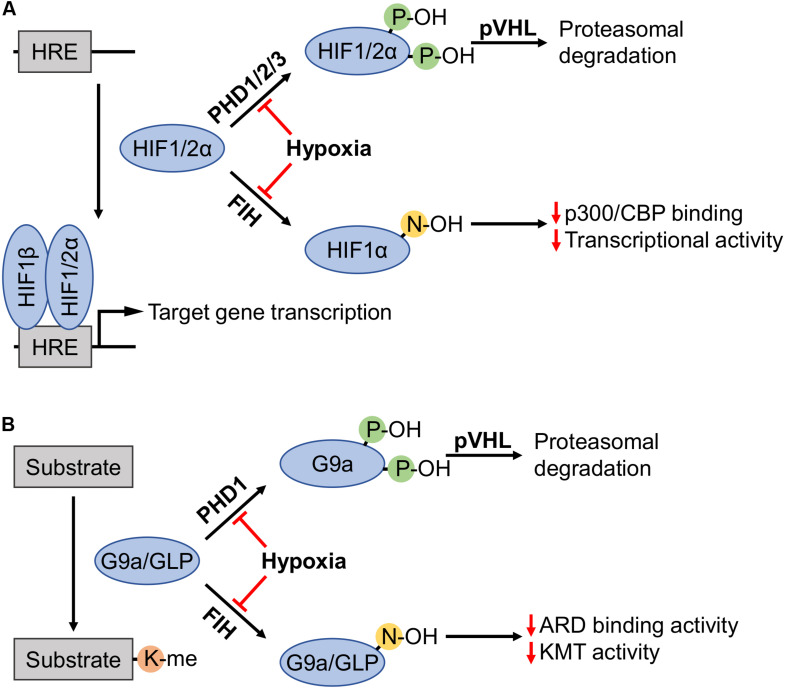
Regulation of HIFα subunits, as well as G9a and GLP by prolyl and asparaginyl hydroxylation. **(A)** Prolyl hydroxylases (PHDs) in the presence of sufficient oxygen levels amenable to enzymatic activity (i.e., normoxia) hydroxylate two proline residues on hypoxia-inducible factor (HIF) α subunits (HIF1α: P402 and P564; HIF2α: P405 and P531). Hydroxylated HIFα subunits are recognized by von Hippel-Lindau tumor suppressor protein (pVHL), recruiting an E3 ubiquitin ligase complex that targets these subunits for proteasomal degradation. Factor-inhibiting HIF (FIH) hydroxylates HIF1α-N803 to inhibit interaction with the p300/CBP coactivators thereby attenuating HIF1α transcriptional activity. Hypoxia inhibits PHDs and FIH, enabling non-hydroxylated HIFα subunits to activate transcription by binding with HIF1β on promoters with hypoxia response elements (HREs). **(B)** G9a and G9a-like protein (GLP) catalyze lysine methylation of histone and non-histone substrates. Like HIFα regulation, PHD1 induces prolyl hydroxylation of G9a (P676 and P1207, occurring at higher stoichiometry at the former proline) and pVHL-mediated proteasomal degradation. FIH hydroxylated ankyrin repeat domains (ARDs) of G9a-N779 and GLP-N867 become impaired in both the ability to bind mono- and dimethylated H3K9 products, and the hydroxylation inhibits di- and trimethylation of H3K9.

The lysine methylation activity exhibited by KMTs is inherent to the Su(var)3-9–Enhancer of zeste–Trithorax (SET) domain, which functions to transfer methyl groups from the methyl donor *S*-adenosyl-L-methionine (SAM) to the ε-amino group of lysine residues (Hyun et al., 2017). Both G9a (KMT1C) and G9a-like protein (GLP, also known as KMT1D) methyltransferases contain SET domains that catalyze mono- and dimethylation of Histone 3 (H3) at the K9 residue (i.e., H3-K9me1/2) (Shinkai and Tachibana, 2011; Shankar et al., 2013). The activity of these KMTs has primarily been attributed to mono- and dimethylation, however, there is evidence for trimethylation activity and specificity differs from other KMTs (Patnaik et al., 2004; Collins et al., 2005; Tsusaka et al., 2018; Al Temimi et al., 2020). Recent reports have demonstrated G9a and GLP to be hypoxia-inducible at the post-translational level, strikingly similar to the regulation of HIFα subunits (Figure 1A; Lando et al., 2003; Schofield and Ratcliffe, 2004; Kaelin, 2005). Given the hypoxia-inducibility of these KMTs, their extensive enzyme-substrate networks, as well as the involvement and regulation of their substrate proteins in hypoxia, the role of G9a in hypoxia may be larger than what is currently known. Aiming to narrow this knowledge gap, we review the functional consequences of these methylation events and the hypoxic nature of these proteins.

## G9a and GLP as Hypoxia Inducible Lysine Methyltransferases

### Regulation via Prolyl Hydroxylation

Similar to the α-subunits of the HIF complexes, multiple studies reported that G9a accumulation in hypoxia is not connected to an increase in its transcription (Chen et al., 2006; Casciello et al., 2017). Interestingly, in mammalian cell lines, hypoxia and dioxygenase inhibitors were both found to increase G9a protein level, accompanied by increased G9a-regulated H3-K9me2 levels (Chen et al., 2006). Although dioxygenase inhibitors are also candidate disruptors of Jumonji C (JmjC)-domain-containing KDM activity that could antagonize this methylation event, it is at least clear that G9a protein level was negatively regulated by an Fe(II)/2-oxoglutarate (2-OG)-dependent dioxygenase. The hypoxic upregulation of G9a was later directly attributed to the reduced hydroxylation of G9a at P676 and P1207 residues (i.e., G9a-P676OH and G9a-P1207OH) by HIF prolyl hydroxylase 1 (PHD1, a.k.a. EGLN2) (Casciello et al., 2017). PHD1 belongs to a group of the most established cellular oxygen sensors (e.g., HIF1α hydroxylases), denoted as such and implicated in hypoxia signaling due to their catalytic requirement for O_2_. Hydroxylation, which occurred more readily at the P676 residue, promotes interaction with the von Hippel-Lindau tumor suppressor protein (pVHL) resulting in downstream ubiquitination and degradation of G9a (Figure 1B). Moreover, the same study demonstrated that a hypoxia-responsive accumulation of G9a was absent in renal cell carcinoma (RCC4) cells deficient in pVHL, but the degradation of G9a could be re-established upon replenishment of wild-type pVHL protein. Therefore, it is speculated that prolyl hydroxylation of G9a occurs over a range of cell types (e.g., lung, breast, and renal cancer), and pVHL is required to trigger degradation. In comparison to the efficiency of HIF1α degradation by the PHD/pVHL pathway across all reported cell types, an appreciable amount of G9a protein was consistently observed in normoxic cells (Chen et al., 2006; Casciello et al., 2017). In addition to cancer cells, activation of G9a by hypoxia has also been observed in human embryonic kidney (HEK293) and mouse embryonic stem (MES) cells. However, when monitoring G9a activity via H3-K9me2 level an increase in methylation was not completely independent of other factors such as KDM inhibition (Chen et al., 2006).

### Regulation via Asparaginyl Hydroxylation

Just as factor-inhibiting HIF (FIH) asparaginyl hydroxylase activity exerts inhibition of HIF1 transcriptional activity in a manner that is independent of protein stability, FIH induces G9a-N779OH and GLP-N867OH proteoforms (Kang et al., 2018; Figure 1B). Interestingly, these conserved residues fall within the methyllysine binding ankyrin repeat domains (ARDs) of both proteins. The asparagine hydroxylated proteoforms display inhibited activity in the context of dimethylation and trimethylation of histone H3, specifically H3K9me2/3. Mechanistically, this likely relates to an interplay between methyllysine binding activity of the ARD and KMT activity of the SET domain. However, it has been shown that mutations which impair ARD methyllysine binding activity of G9a protein have no effect on *in vitro* KMT activity (Collins et al., 2008).

**FIGURE 2:**
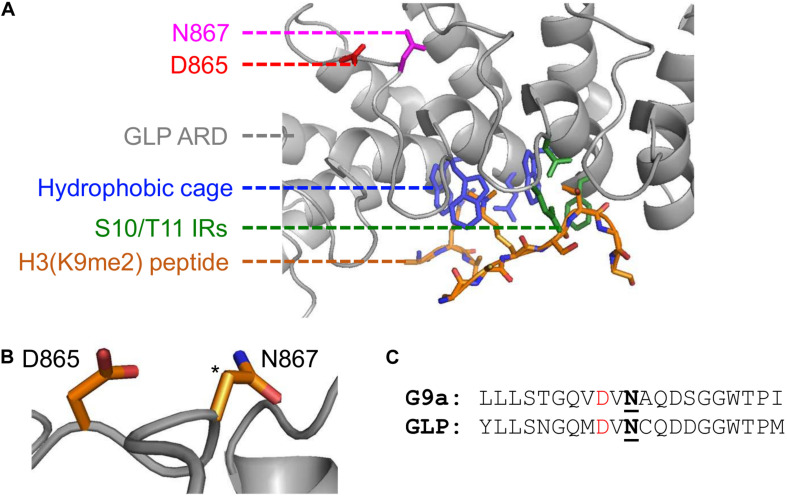
Implications of asparaginyl hydroxylation within the ARDs of G9a and GLP methyltransferases. **(A)** Crystal structure of G9a-like protein (GLP) ankyrin repeat domain (ARD) domain in complex with dimethylated H3 N-terminal tail visualized with PyMOL (PDB ID, 3B95; Collins et al., 2008). Binding of a dimethylated peptide (orange backbone) is mediated by the hydrophobic binding cage (blue) and H3-S10/T11 interacting residues (IRs; green) of the GLP ARD (white, cartoon representation). The GLP(N867) hydroxylation site (pink) is distant from the peptide binding region and is adjacent to the D865 residue (red). **(B)** The proximity of the D865 and N867 residues, where the target hydroxylated atom (i.e., β-carbon of N867) is denoted by an asterisk. **(C)** Sequence similarity between G9a and GLP asparaginyl hydroxylated regions, up- and downstream ten residues from the modified asparagine (bold, underlined). Candidate hydrogen bonding aspartates (red) occur two residues upstream the G9a-N779 and GLP-N867 hydroxylation sites.

Computationally, Kang and colleagues demonstrated that hydroxylation destabilizes the ARD-H3K9me2 interaction by disrupting a structural pocket that facilitates methyllysine binding. It is well established that the ARDs within G9a and GLP mediate binding to H3K9me1/2 through a hydrophobic cage consisting of three tryptophan residues and one acidic residue (Collins et al., 2008). However, the GLP-N867 hydroxylation site is spatially distant from the hydrophobic binding cage (Figure 2A). Noteworthy, FIH asparaginyl hydroxylation activity extends to ARDs within numerous other proteins and is reviewed by Cockman et al. (2009). Although the conformation of many ARDs does not appear to be affected by asparagine hydroxylation when analyzed in crystal structure, in solution a hydrogen bond can be established between the introduced hydroxyl group and an adjacent aspartyl residue (2 residues upstream from the hydroxylation site) (Coleman et al., 2007; Kelly et al., 2009). From the GLP crystal structure, this potential hydrogen bonding interaction is likely as the N867 β-carbon is directly positioned toward the oxygen of the D865 side chain (Figure 2B). Additionally, within the G9a primary structure this D-N pairing is also present in the context of the N779 hydroxylation site (Figure 2C). Whether this D-N-OH hydrogen bonding occurs in the context of G9a and GLP methyltransferases and how it may lead to the opening of the hydrophobic cage remains to be determined.

## G9a- and GLP-Dependent Non-Histone Protein Methylation

### Lysine Methylation as a Signaling Mechanism for Cellular Hypoxia Adaption

In the same manner as the HIF1α hydroxylases, the catalytic requirement for O_2_ is inherent to other Fe(II)/2-OG-dependent dioxygenases, such as JmjC KDMs (Batie and Rocha, 2019). It is well-established that any loss of JmjC KDM activity, or any Fe(II)/2-OG-dependent dioxygenase, is more complex than just the loss of dioxygen. The catalytic activity of JmjC KDMs is also specifically tied to the individual affinities for molecular oxygen (K_*m;O2*_), in addition to other metabolic factors that are inherent to metabolism in the hypoxic environment (Chang et al., 2019). Recently, KDM5A and KDM6A have been established as cellular oxygen sensors that display notably high K_*m;O2*_ values, such that the inhibition of these KDMs in hypoxia is comparable to that of the HIF1α hydroxylases (Batie et al., 2019; Chakraborty et al., 2019). It has also been demonstrated that KDMs with amine oxidase activity, such as lysine-specific demethylase 1 (LSD1), display reduced activity in prolonged hypoxia. This is the result of reduced availability of the cofactor flavin adenine dinucleotide (FAD) in the hypoxic environment (Yang et al., 2017). Nonetheless, extreme oxygen deprivation (e.g., prolonged hypoxia or anoxia) would be anticipated to abolish the normal level of JmjC activity. Such an environment would change the opposing balance between normal KMT and KDM activity and set the stage to promote KMT-driven methylation events. In other words, as the catalytic mechanism of KMTs is independent of oxygen, hypoxia may exist as a contextual switch for KMT-driven effects to manifest over KDM-driven effects.

As G9a and GLP are hypoxia-inducible, the KMT activity of these enzymes may contribute novel molecular inputs that shape the cellular adaptive response to hypoxia. Within the realm of KMTs with known non-histone substrates, G9a has a well-established and relatively numerous substrate network, second only to SETD7 (Biggar et al., 2017). Furthermore, the biological roles of protein-modifying enzymes may be directly attributed to that of their modified substrate(s). Therefore, the following sections focus on; (1) describing G9a and GLP-driven non-histone lysine methylation sites, (2) discussing the known biology of the methylation sites or residue, and (3) whether the substrates have any known function regarding hypoxia response. It is crucial to understand the implications of G9a and GLP non-histone methylation events and the known function of these substrate proteins in the hypoxia response.

### HIF1α Methylation

**FIGURE 3:**
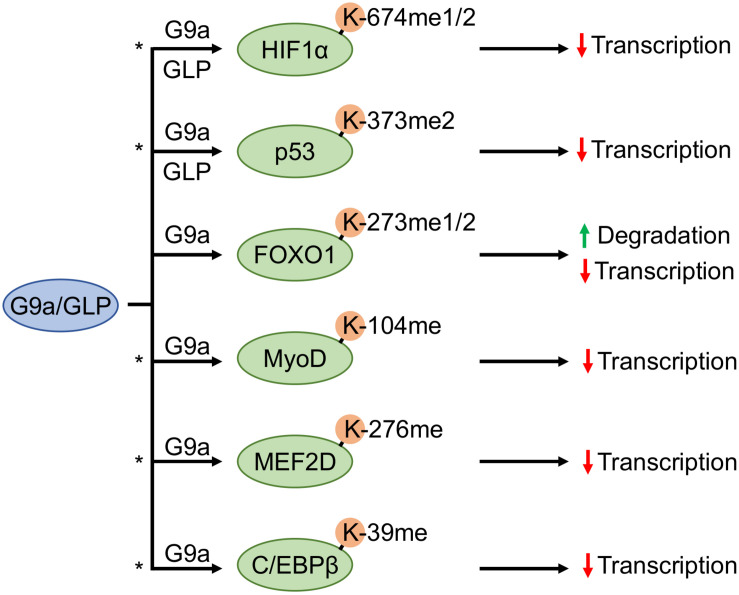
G9a and GLP methyltransferases antagonistically regulate substrate transcription factors. G9a and G9a-like protein (GLP) facilitate methylation of hypoxia inducible factor 1α (HIF1α) and tumor suppressor protein p53, whereas forkhead box O1 (FOXO1), myoblast determination protein 1 (MyoD), myocyte enhancer factor 2D (MEF2D), and CCAAT/enhancer-binding protein-beta (C/EBPβ) methylation have only been shown to occur by G9a. An asterisk denotes methylation events that may participate in a methylation-acetylation switch at the same residue (based upon known acetylation events of the same protein, or in the case of MEF2D an acetylation event in conserved regions on other MEF2 family members).

Since its discovery, HIF1α has been positioned as the master regulator of cellular response to hypoxia. HIFs enable adaption to low oxygen through the upregulation of gene expression programs that drive physiological changes; including, metabolic reprogramming and vascularization (Krock et al., 2011; Courtnay et al., 2015). In the last decade, lysine methylation has been shown to participate in the complex combinatorial PTM code that regulates HIF1α function. Specifically, three methylated proteoforms (HIF1α-K32me1, HIF1α-K391me2, and HIF1α-K674me1/2) have demonstrated methylation-dependent functional consequences in the context of promotor occupancy, stability, and transcriptional activity (Liu et al., 2015; Kim Y. et al., 2016; Lee et al., 2017; Bao et al., 2018). More recently, the HIF1α-K674me1/2 proteoform demonstrates altered transcriptional activity by consequence of G9a and GLP methylation. Bao and colleagues studied G9a and GLP catalyzed induction of the HIF1α-K674me1/2 proteoform within the context of glioblastoma cell migration. The biochemical characterization of both the interaction and functional consequences were performed in HeLa cells. Interestingly, the methylation of HIF1α-K674 by G9a and GLP also occur in glioblastoma cell lines and suppress HIF-1-dependent migration. Methylation of this site was shown to occur in an oxygen-independent manner. Albeit, this was demonstrated under conditions with overexpressed exogenous wild-type and mutant G9a proteins. However, the HIF1α-K674me1/2 proteoform does demonstrate reduced transcriptional activity in the absence of either reduced protein stability or DNA binding activity (Bao et al., 2018; Figure 3).

The biochemistry of the HIF1α-K674 residue is known to influence transcriptional activity. The HIF1α-K674ac acetylated proteoform is more transcriptionally active due to increased association with the p300 co-activator, thus a PTM-blockage of acetylation would introduce the possibility of decreased recruitment (Lim et al., 2010). Interestingly, Bao and colleagues demonstrated that within glioblastoma cells methylation of this residue or mutation to arginine did not alter promotor occupancy of p300 nor the binding of p300 and other co-regulators to HIF1α (Bao et al., 2018). However, methylation does negatively regulate HIF1α transcriptional activity, target gene expression, and glioblastoma multiforme cell migration. Therefore, the mechanism whereby G9a and GLP-catalyzed HIF1α-K674 methylation impairs HIF1α transcriptional activity remains elusive in glioblastoma cell lines. As the preliminary work documenting the cellular consequence of K674 acetylation status did demonstrate reduced HIF1α interaction with p300 by mutation of this residue to arginine, it would be anticipated that this discrepancy may be inherent to differences between these cell types (Lim et al., 2010).

### Non-HIF Transcription Factors and the Hypoxia Response

The regulation of HIF1α by G9a- and GLP-dependent lysine methylation of the K674 residue has wide implications on hypoxic gene expression programs due to the capacity to which HIF1α controls cellular hypoxic adaption. These KMTs also exert regulation of other transcription factors, implicating them in a larger array of transcriptional programs through their non-histone KMT activities (Figure 3).

#### p53

The tumor suppressor protein p53 is a target of both G9a and GLP, as these enzymes induce the p53-K373me2 proteoform (Huang et al., 2010). The p53-K373me2 proteoform appears to be inactive regarding the ability to drive apoptosis, which is rescued upon depletion of these KMTs. Therefore, Huang and colleagues have described G9a and GLP as putative oncogenes due to both their effect on p53 function and that they are overexpressed in a variety of cancers. Opposing methylation, acetylation of this residue, and other lysine residues within the C-terminal domain, promote p53 activity (Gu and Roeder, 1997; Luo et al., 2004; Ivanov et al., 2007). Apart from the role of p53 in DNA damage response, p53 also responds to other cellular cues such as hypoxia (Hammond and Giaccia, 2005; Sermeus and Michiels, 2011). Briefly, the hypoxic p53-response differs from the typical p53-response (i.e., induction of apoptosis through upregulation of pro-apoptotic gene transcription). Most evidence demonstrates hypoxia to induce a p53 trans-repressor function, with little evidence for transactivation activity, however, apoptosis is still the major outcome of hypoxia p53 signaling (Hammond and Giaccia, 2005). For example, a trans-repressor function of p53 has been demonstrated for the *survivin* (i.e., *BIRC5*) gene, an inhibitor of apoptosis (Hoffman et al., 2002; Mirza et al., 2002). More specifically, whether p53 signaling in the hypoxic state drives apoptosis appears to depend on the degree of oxygen restriction (i.e., oxygen concentration and duration) in a dynamic and reciprocal interplay (e.g., competition for co-activators, direct and indirect interactions with regulators within respective protein-protein interaction (PPI) networks, etc.) with HIF1α (Hammond and Giaccia, 2005; Sermeus and Michiels, 2011).

#### FOXO1

Forkhead box O1 (FOXO1) belongs to a family of transcription factors known to have tumor suppressor roles in a wide variety of cancers. Numerous signaling cascades impinge upon FOXO1, relevant to a wide variety of normal biological and pathological contexts (Kandula et al., 2016; Xu et al., 2017; Peng et al., 2020). As a well-documented example, insulin triggers the PI3K-PKB signaling cascade that leads to phosphorylation of FOXO1 (pFOXO1) and subsequent proteasomal degradation (Matsuzaki et al., 2003).

FOXO1 is regulated by a variety of PTMs that affect transcriptional activity, DNA binding activity, and protein stability (Matsuzaki et al., 2003, 2005; Daitoku et al., 2004; Huang et al., 2005). Regarding protein stability, proteasomal degradation of FOXO1 occurs via ubiquitination by SKP2, an E3 ubiquitin ligase (Huang et al., 2005). Recently, G9a methylation of FOXO1 at K273 was shown to be a novel molecular-input that potentiates the interaction between SKP2 and FOXO1 (Chae et al., 2019). In colon cancer, G9a-induced proteasomal degradation of FOXO1-K273me1/2 proteoforms enhances colon cancer cell proliferation and colony formation.

Digestive malignancies are a well-reviewed cancer type by which FOXO1 exerts a tumor-suppressive role (Shi et al., 2018). Specifically, FOXO1 promotes apoptosis and is inhibitory of proliferation, differentiation, and angiogenesis. Hypoxia-induced angiogenesis is beneficial for the progression of certain pathologies, such as tumor growth by increasing the blood supply to malignant cells (Krock et al., 2011). In gastric cancer it is clear that FOXO1 functions to inhibit angiogenesis, and gastric carcinomas with pFOXO1 (inactive proteoform) are associated with higher expression of angiogenic drivers such as HIF1α and vascular endothelial growth factor (VEGF) (Kim et al., 2011; Kim S. Y. et al., 2016). Thus, this introduces the possibility of G9a contributing toward the degradation of FOXO1 to further promote hypoxia-induced angiogenesis. Albeit, this would depend on whether the G9a-FOXO1 interaction identified in colon cancer holds true in the context of FOXO1-inhibition of angiogenesis in gastric cancer.

#### C/EBPβ

CCAAT/enhancer-binding protein-beta (C/EBPβ) belongs to a family of bZIP transcription factors (Tsukada et al., 2011). These transcription factors regulate transcriptional programs required for normal cell function, as well as functions closely associated with pathological processes such as tumorigenesis (Nerlov, 2007). G9a catalyzes methylation of C/EBPβ (K39 in mouse, K43 in humans) within the transactivation domain (Pless et al., 2008). Functionally, this methylation inhibits the transactivation activity of C/EBPβ, and a methylation deficient mutant further augments C/EBPβ target gene expression. Although there is a discrepancy between the effect of K39 methylation-null mutants between Pless and colleagues and a complementary study investigating K39 acetylation, as p300-dependent acetylation at this site has been shown to enhance C/EBPβ activity (Ceseña et al., 2007). Nonetheless, G9a acts as a repressor of C/EBPβ-dependent transcription through direct methylation and likely antagonistically competes with acetylation to modulate C/EBPβ activity. It is unclear whether reduced transactivation is due to decreased C/EBPβ DNA binding or promotor occupancy of the methylated C/EBPβ proteoform and its associated factors. However, hypoxia has been shown to inhibit C/EBPβ DNA binding activity during adipogenesis (Park and Park, 2010).

#### MEF2D

Myocyte enhancer factor 2D (MEF2D) protein belongs to the family of MEF2 transcription factors that regulate biological processes in skeletal muscle (Estrella et al., 2015). In myocyte differentiation, different members of the MEF2 family regulate distinct gene expression programs, and specifically MEF2D controls expression of genes responsive to Janus kinase 2 (JAK2)-like signaling and hypoxia signaling (Estrella et al., 2015). Furthermore, dysregulation of the MEF2 family has important implications in tumorigenesis (Di Giorgio et al., 2018). In colorectal cancer, MEF2D is a target gene of HIF1α, and MEF2D enhances transcriptional upregulation of proangiogenic cytokines (Xiang et al., 2017).

Within the context of myocyte differentiation, the transcriptional activity of MEF2D is modulated by the lysine methylation status of residue K267. This methylation site is controlled by the opposing activities of G9a and LSD1 (Choi et al., 2014). Methylation-deficient mutant MEF2D-K267R binds greater to the *Myogenin* promoter (i.e., a MEF2D target gene) than the WT protein. Such findings indicate a possible functional role for G9a methylation at this site in reducing transcriptional activity by attenuating the amount of chromatin-bound MEF2D. Furthermore, the authors postulated that DNA-binding activity may be regulated by a methylation-acetylation switch at this residue, as MEF2C is acetylated by p300 acetyltransferase at several conserved sites, where one of which is a conserved site with MEF2D-K267 (Ma et al., 2005).

#### MyoD

The inhibitory mechanisms exerted by G9a on skeletal myocyte differentiation also extends toward transcriptional programs controlled by myoblast determination protein 1 (MyoD) (Ling et al., 2012a). Like MEF2D, MyoD is a transcription factor controlling gene expression programs that regulate fundamental biological processes in skeletal muscle, including myogenesis and differentiation (Sartorelli and Caretti, 2005; Tapscott, 2005). Hypoxia is known to inhibit skeletal muscle differentiation. During periods of myoblast hypoxia, MyoD degradation is accelerated and thereby reduces the expression of MyoD target genes that drive differentiation (Di Carlo et al., 2004).

Ling et al. (2012a) first demonstrated that the catalytic activity of G9a inhibited differentiation, and correspondingly differentiation was enhanced by either the reduction or inhibition of G9a protein. This was primarily attributed to methylation of MyoD-K104 by G9a, and this methylation occurred more readily in undifferentiated cells to block differentiation by reducing transcriptional activity. The interaction is facilitated by an adaptor protein, Sharp-1, and repressive chromatin methylation may also occur (Azmi et al., 2004; Ling et al., 2012b). The mechanism by which MyoD-K104 methylation directly inhibits transcriptional activity is not yet clear, however, the collective acetylation of three neighboring lysines (K99, K102, and K104) by p300/CBP-associated factor (PCAF) acetyltransferase likely induces an allosteric change in conformation that increases MyoD DNA binding affinity (Sartorelli et al., 1999; Ling et al., 2012a). Therefore, G9a-driven methylation of MyoD-K104 may function to antagonistically regulate acetylation, preventing MyoD-controlled differentiation.

Overall, G9a provides another molecular-input to inhibit MyoD function. Given the recently found hypoxia-inducible nature of G9a, and that MyoD is negatively regulated by hypoxia through changes in stability, G9a may present another plausible avenue by which hypoxia exerts regulation of MyoD function.

### Methylation of Chromatin Remodelers

#### Reptin and Pontin

**FIGURE 4:**
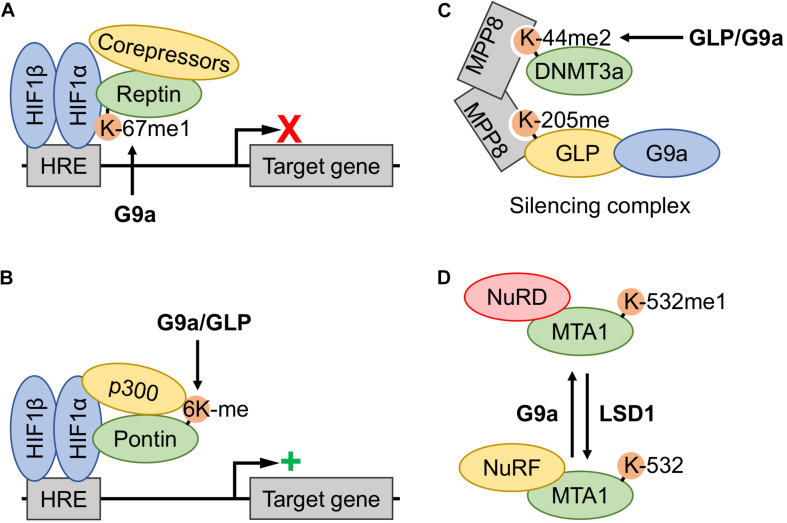
Involvement of G9a and GLP methyltransferase activity in the functional regulation of other transcriptional regulators. **(A)** Hypoxia induced G9a monomethylation of Reptin-K67 increased association with the hypoxia-inducible factor (HIF)-1 complex, thereby downregulating expression of a subset of the hypoxia response element (HRE)-controlled target genes by recruitment of corepressors. **(B)** Hypoxia induced methylation of Pontin at six lysine residues (6Kme) is catalyzed by both G9a and G9a-like protein (GLP), thereby increasing recruitment of the p300 coactivator to the HIF-1 complex at a subset of HRE-containing promotors and augmenting target gene transcription. **(C)** The DNA methyltransferase 3a (DNMT3a) (mouse K44me2, human K47me2) proteoform assembles into a DNMT3a-MPP8-GLP/G9a silencing complex, where the DNMT3a methylation site acts as a docking site for the MPP8 chromodomain. Both GLP and G9a may induce methylation, however, the former is believed to be the primary KMT. **(D)** Metastasis-associated protein 1 (MTA1)-K532 methylation status dictates association with nucleosome remodeling and deacetylase complex (NuRD) and nucleosome remodeling factor (NuRF) complexes. G9a monomethylation of K532 promotes MTA1 nucleation of the NuRD corepressor complex, whereas unmethylated or demethylated (lysine-specific demethylase 1 (LSD1)-dependent) proteoforms nucleate the NuRF coactivator complex.

Reptin and Pontin are two members of multimeric chromatin remodeling complexes known to regulate transcription of pathways that are relevant to cancer (Huber et al., 2008). Interestingly, both proteins are involved in hypoxia signaling through the HIF1 pathway, and this signaling pathway is, in part, regulated by G9a-dependent lysine methylation (Lee et al., 2010, 2011). The methylation events were hypoxia-inducible as G9a protein level was increased in the cell types used (G9a: MCF7, MEFs, HeLa, and ES cells; GLP: HeLa and MCF7) (Lee et al., 2010, 2011). In this model, the hypoxia-induced Reptin-K67me1 proteoform exerts repression of the transcription of a subset of hypoxia-related genes, including *VEGF* (Lee et al., 2010). Mechanistically, the Reptin-K67me1 proteoform has enhanced interaction with HIF1α and recruits corepressors such as histone deacetylase 1 (HDAC1) (Figure 4A).

In the case of Pontin, G9a and GLP were shown to catalyze methylation on multiple lysine residues (K265, K267, K268, K274, K281, and K285) (Lee et al., 2011). Accordingly, both the protein levels of G9a and GLP, as well as the methylation status of Pontin, were increased in hypoxia. In a model that is reflective of Reptin function under hypoxia, Pontin associates with the HIF1 complex at the promotors of HIF1 target genes. However, methylated Pontin proteoforms enhance the recruitment of the p300 coactivator and was found to increase HIF1 transcriptional activity (Figure 4B).

#### CSB

In screening the substrate selection of G9a, Rathert et al. (2008) demonstrated Cockayne syndrome group B (CSB) protein to be methylated at four residues (K170, K297, K448, and K1054) using peptides that represent these sites *in vitro*. Supporting this model, both G9a and CSB are known to interact with each other (Yuan et al., 2007). The functional consequence of CSB methylation is unknown at this time, however, the gene encoding the CSB protein is a target of HIF1 and it is a critical factor in prompting a cellular response to hypoxia (Filippi et al., 2008). CSB functions to distribute the p300 coactivator between p53 and HIF1α transcription factors, as CSB competes with p300 for binding to p53 (Filippi et al., 2008; Ye et al., 2019). Therefore, the CSB protein is involved in regulating the interplay between HIF1α and p53 transcription factors and regulating the activities of either protein in a hypoxia-dependent manner (Filippi et al., 2008; Frontini and Proietti-De-Santis, 2009; Vélez-Cruz and Egly, 2013; Ye et al., 2019).

### Methylation of DNA Methyltransferases

#### DNMT3a

G9a and GLP have been reported to methylate DNA methyltransferases (DNMTs). G9a and GLP KMTs have been demonstrated to mediate the dimethylation of mouse DNMT3a-K44 both *in vitro* and in tissue culture (Chang et al., 2011). Biochemical experiments have demonstrated that the DNMT3a-K44me2 proteoform (equivalent to human DNMT3a-K47me2) is a member of a silencing complex via interaction of this methyl-modification with the methyllysine binding chromodomain of MPP8 (Figure 4C). This methyl-dependent complex consists of a MPP8 dimer, whereby the chromodomain in each monomer facilitates interaction with either methylated DNMT3a or GLP. Thus, GLP induces the formation of the DNMT3a-K44me2 proteoform, which is recruited into a complex consisting of DNMT3a-MPP8-GLP/G9a; a complex known to repress gene expression through methylation of chromatin at the histone and DNA level.

In healthy cells, DNMT3a functions as a suppressor of cellular adaption to hypoxia by negatively regulating the HIF2α-driven oxygen-sensing pathway; regulation that is mediated through epigenetic silencing of the *EPAS1* gene (encoding the HIF2α protein) via DNA methylation (Lachance et al., 2014). Activation of *EPAS1* expression plays a central role in driving aggressive tumor phenotypes such as proliferation, angiogenesis, metastasis, and differentiation (Qing and Simon, 2009). Lachance and colleagues demonstrated that the proliferation of cancer cells in a hypoxic microenvironment is driven by activation of the HIF2α pathway; resulting from naturally occurring defects in DNMT3a (Lachance et al., 2014). Wildtype DNMT3a has a tumor-suppressing role through preventing HIF2α-dependent hypoxic cancer cell proliferation. Whether this DNMT3a-MPP8-GLP/G9a complex, another DNMT3a constituted function, or DNMT3a alone is responsible for the silencing of the *EPAS1* gene is unknown. However, G9a and GLP induce the formation of the DNMT3a-MPP8-GLP/G9a silencing complex through DNMT3a-K44me2 (mouse; K47me2, human) methylation and therefore may facilitate the tumor-suppressive role of DNMT3a (Chang et al., 2011; Lachance et al., 2014).

Furthermore, Rathert and colleagues reported DNMT1(K70) as an *in vitro* non-histone target of G9a-mediated methylation, and it should be noted that G9a and DNMT1 are also known to have a physical interaction (Estève et al., 2006; Rathert et al., 2008).

### Nucleosome Remodeling and Deacetylase Complex

The nucleosome remodeling and deacetylase complex (NuRD) interacts with transcription factors to dictate local gene accessibility and modulate the histone PTM landscape, thereby regulating transcription (Denslow and Wade, 2007). Thus far, evidence exists for the G9a catalyzed lysine methylation of two known NuRD complex members (i.e., HDAC1 and MTA1). The NuRD complex imposes regulatory control over HIF1α protein function and other transcription factors; therefore, these G9a substrates, the biochemistry of each targeted residue, and involvement of these proteins in HIF1α regulation are discussed in the following section.

#### HDAC1

Similar to other histone-regulators, HDAC1 is a component of several co-repressor complexes and is now implicated in multiple biological processes beyond epigenetics due to the discovery of numerous non-histone substrates (Nalawansha et al., 2018). In substrate profiling experiments, the *in vitro* activity of G9a was demonstrated against peptides representing the K432 residue, albeit at a level that was lower than other non-histone sites reported in the study (Rathert et al., 2008). Furthermore, HDAC1 and G9a form physical interactions with each other to mediate silencing of transcriptional programs by the synergistic induction of H3-K9 methylation (Duan et al., 2005; Wang et al., 2008, 2017). Although the functional consequence of K432 methylation of this is unknown, and efforts should be made to validate this in cell models, K432 acetylation status directly dictates deacetylase activity (Qiu et al., 2006; Luo et al., 2009). The p300 acetyltransferase induces acetylation of six lysyl residues, whereby acetylation of K432 has been shown to inactivate deacetylase activity (Qiu et al., 2006). Well-documented models of the functional interplay between methylation and acetylation are known to occur within both p53 and RelA proteins, albeit on distinct neighboring residues (Ivanov et al., 2007; Kurash et al., 2008; Yang et al., 2010). Currently observed within the histone-code, methylation and acetylation of the same lysyl residue exist in a mutually exclusive manner, therefore it would be anticipated that HDAC1-K432 methylation and acetylation are antagonistic to each other. Whether HDAC1-K432me and HDAC1-K432ac proteoforms function synergistically or antagonistically regarding the level of inherent deacetylase activity is unknown, however, it is established that the biochemical properties of this residue are critical in influencing HDAC1 protein function.

Within the context of HIF signaling, multiple deacetylases exert regulatory control over HIF1α and HIF2α function by modulating PPIs, protein stability, and activity. Moreover, HDAC inhibitors have been demonstrated to promote HIF1α protein stability, and HDAC1 and HDAC3 were shown to enhance stability and bind to the oxygen-dependent degradation domain (ODDD) of HIF1α (Kim et al., 2007). Specifically, HDAC1 deacetylase activity antagonizes HIF1α-K532ac and HIF1α-K709ac proteoforms (Yoo et al., 2006; Geng et al., 2012). Acetylation of both these sites has opposing effects on HIF1α protein stability, where the former proteoform has reduced stability and the opposite is observed of the latter. Therefore, HDAC1 deacetylase activity is a regulator of HIF1α stability through these acetylation sites. Given that G9a and GLP methyltransferases directly regulate HIF1α by induction of HIF1α-K674me1/2 proteoforms, it would be interesting to see if G9a exerts control over of HIF1α acetylation status through modulation of HDAC1 activity.

#### MTA1

Metastasis-associated protein 1 (MTA1) works in conjunction with HDAC1 to promote HIF1α protein stability, mediated by the deacetylation of the unstable HIF1α-K532ac proteoform (Yoo et al., 2006). MTA proteins, specifically MTA1, are present within the NuRD complex and physically interact with HDACs (Xue et al., 1998; Yao and Yang, 2003). In this manner, MTA1 enhances the deacetylation of HIF1α at the K532 site by mediating the interaction between HDAC1 and acetylated HIF1α. In this model, the NuRD complex (or at least its MTA1/HDAC1 sub-components) acts as a co-activator of HIF1α, whereas in other cases the NuRD complex is a known co-repressor (Mazumdar et al., 2001; Yan et al., 2003).

In delineating the paradoxical co-regulatory role of MTA1, Nair and colleagues demonstrated that the methylation status of MTA1-K532 acted as a molecular switch for association with co-activator and co-repressor complexes. This modification was also dynamically regulated by the opposing action of methyl-regulator proteins; LSD1 and G9a (Nair et al., 2013; Figure 4D). G9a was determined to induce the formation of the monomethylated MTA1-K532me1 proteoform, promoting the assembly of a repressive NuRD complex. LSD1 was found to remove this modification, and LSD1-catalyzed demethylation or methylation-null MTA1-K532R triggered a coactivator role for MTA1. Specifically, demethylated MTA1 associated with the nucleosome remodeling factor (NuRF) complex to trigger activation of gene expression via LSD1-dependent demethylation of repressive H3K9me2 and subsequent H3-K9 acetylation by p300/CBP.

Therefore, G9a KMT activity directly facilitated the repressive activity of the NuRD complex through induction of the MTA-K532me1 proteoform (Nair et al., 2013). Whether this methylation event is enhanced in hypoxia due to G9a activation is unclear, however, it is relevant to consider that LSD1 demethylase activity has been demonstrated to be inhibited in prolonged hypoxia due to decreased cellular availability of the flavin adenine dinucleotide cofactor (Yang et al., 2017).

### Other Non-histone Substrates

It is clear that G9a and GLP post-translationally modulate the functions and fate of numerous proteins that are relevant to hypoxia signaling. Indeed, the KMT activity of G9a also extends to Sirtuin 1 (SIRT1) K662 both *in vitro* and in tissue culture (Moore et al., 2013). SIRT1 is tightly involved in hypoxia response as this deacetylase directly modifies HIF1α and HIF2α transcription factors and in doing so modulates protein function (Dioum et al., 2009; Lim et al., 2010; Laemmle et al., 2012; Yoon et al., 2014; Joo et al., 2015). Albeit, the functional consequence of this methylation event and the biology of the K662 residue are unknown. Moreover, G9a and GLP catalyze lysine methylation of several other non-histone substrates, for which little is known regarding their hypoxic post-translational regulation or the involvement of these proteins in the hypoxic landscape. These include chromodomain Y-like protein (CDYL1), widely-interspaced zinc finger-containing protein (WIZ), apoptotic chromatin condensation inducer in the nucleus (ACINUS), krueppel-like factor 12 (KLF12), activating transcription factor 7-interacting protein 1 (ATF7IP), and DNA ligase 1 (LIG1); whereby a number of these examples have altered function imparted as a consequence of G9a-directed methylation (Rathert et al., 2008; Ferry et al., 2017; Tsusaka et al., 2018). The hypoxia-inducibility of G9a activity may present a link whereby hypoxia may regulate the functions of these proteins. Furthermore, G9a and GLP themselves both contain reciprocal self/auto-methylation sites, several of which dictate the function of these enzymes (Chin et al., 2007; Rathert et al., 2008; Chang et al., 2011).

## Hypoxic Methylation and Pathological Relevance

**FIGURE 5:**
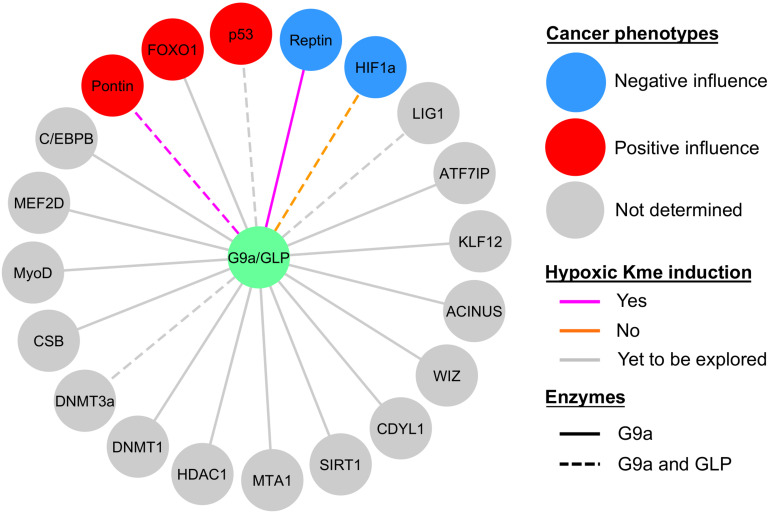
Current knowledge of G9a and GLP non-histone substrate modification in hypoxia and relevance to cancer biology. Sphere color denotes whether the effect of substrate methylation on cancerous properties (e.g., tumor growth, proliferation, degree of apoptosis, migration, etc.) was negative (blue circles), positive (red circles), or not determined (gray circles). Methylation (Kme) of Reptin and Pontin were hypoxia inducible (magenta lines), HIF1α methylation was not induced by hypoxia (orange lines), and hypoxic induction of Kme is yet to be explored in the remaining substrates (gray lines). G9a facilitates methylation of all the substrates shown, whereas evidence for GLP methylation is denoted by dashed lines.

As hypoxia is an inherent feature of solid tumors it is perhaps not surprising that HIF1α plays a major role in the pathophysiology of cancer (Masoud and Li, 2015). In this manner, HIF1 target genes drive pathways and biological processes that promote cancer progression. In comparison, the current knowledge of hypoxia-inducible G9a and GLP non-histone substrate methylation is less complete (Figure 5), but it is clear that lysine methylation plays a key role in affecting cancer pathways (McGrath and Trojer, 2015; Carlson and Gozani, 2016; Clarke et al., 2020). Below, we discuss similar implications regarding the G9a and GLP relevance in cancer progression, such as through hypoxia-responsive non-histone methylation.

To date, G9a is known to be involved in various biological processes (e.g., cancer, synaptic plasticity, lymphoid cell differentiation and function) (Casciello et al., 2015; Sheer and Zaph, 2017; Pang et al., 2019). This KMT plays a role in regulating cancerous phenotypes through histone and non-histone methyltransferase activities, also genetic upregulation is associated with more aggressive phenotypes and poor prognosis (Casciello et al., 2015). Hypoxic upregulation of G9a via inhibition of PHD1 mediates histone H3K9 methylation and silencing of a subset of genes, and inhibition of G9a reduces proliferation, migration, and *in vivo* tumor growth (Casciello et al., 2017). Furthermore, in ovarian cancer hypoxic induction of G9a and GLP activity, via the loss of FIH activity, leads to reduced expression of metastasis-suppressor genes via H3K9 methylation (Kang et al., 2018). Within these contexts, hypoxia may epigenetically promote tumorigenic potentials through these KMTs.

Within the context of hypoxia-inducibility of G9a and GLP, the effector functions exerted by these enzymes are relevant to HIF1-dependent pathological phenotypes via modification of several of the substrates described previously in this review. Hypoxia-induced G9a/GLP-dependent methylation of Pontin enhances HIF1α-dependent transcription of a subset of target genes, such as *Ets1* (Lee et al., 2011). The presence of methylatable wild-type Pontin leads to an increase in breast cancer tumorigenesis, namely in the context of cell proliferation, migration, and invasiveness. However, these KMTs also show evidence for their role in suppressing cancerous phenotypes in the context of Reptin-K67me1 and HIF1α-K674me1/2 methylation. The biological consequence of a deficiency in G9a-dependent Reptin methylation (i.e., a methylation-deficient Reptin-K67R mutant) in breast cancer is enhanced proliferation, migration, invasion, and increased tumor mass (Lee et al., 2010). Furthermore, HIF1α-K674me1/2 methylation by G9a/GLP impairs HIF1-dependent migration of glioblastoma cell lines (Bao et al., 2018). However, in comparison to normal tissues, G9a mRNA and protein expression is lower in glioblastoma and chronic hypoxia leads to downregulation of these KMTs, establishing an anti-correlation relationship between G9a and HIF1-target gene expression (Bao et al., 2018).

Often G9a and GLP activities are described as inhibitory targets for cancer treatment (Casciello et al., 2015; Charles et al., 2019; Milite et al., 2019); for example, Huang and colleagues reported G9a and GLP to be overexpressed in a variety of cancers, denoting them as candidate oncogenes (Huang et al., 2010). Supporting this, a higher G9a expression has been correlated with poor prognosis in terms of clinicopathological features of gastric cancer and poor patient outcomes (Zhang et al., 2019). Clearly, modulating G9a expression and activity has potential as a therapeutic strategy. Further, increased self/auto-methylation of G9a has been found to restore the sensitivity of glucocorticoid-resistant acute lymphoblastic leukemia tumors to glucocorticoid treatment (Poulard et al., 2018). Examples of G9a and GLP inhibitors developed to date include, but are certainly not limited to, BIX-01294 (Kubicek et al., 2007), UNC0638 (Vedadi et al., 2011), UNC0642 (Liu et al., 2013), A-366 (Sweis et al., 2014), and CSV0C018875 (Charles et al., 2020). Modulation of G9a function with chemical inhibitors has presented to be beneficial for sensitization to other standard chemotherapeutics, as described regarding enhancing G9a auto-methylation in combination with glucocorticoid treatment (Poulard et al., 2018). This is the case for direct inhibitors of G9a and GLP methyltransferase activity. For example, BIX-01294 treatment sensitizes human glioma cells to temozolomide (Ciechomska et al., 2018). Increased focus on the application and development of G9a and GLP inhibitors suitable for animal studies, such as UNC0642, will be critical for advancing such therapeutic strategies (Liu et al., 2013).

The development of pharmacological inhibitors of G9a and/or GLP has resulted in a capability to selectively inhibit these KMTs (Charles et al., 2019; Milite et al., 2019). However, caution in the use of G9a inhibition as a therapeutic strategy in cancer has recently been advocated (Seton-Rogers, 2019). Such inhibitors certainly demonstrate promising therapeutic potential, some examples provided for ovarian cancer and non-small cell lung cancer (Zhang et al., 2018; Watson et al., 2019). However, recent studies have also shown that pharmacological inhibition of G9a is detrimental in the context of skin and lung cancer (Avgustinova et al., 2018; Rowbotham et al., 2018; Seton-Rogers, 2019). Although G9a-abalated squamous cell carcinomas have delayed initiation, such tumors are more aggressive, genetically unstable, as well as have selective pressure to inactivate p53 signaling (Avgustinova et al., 2018). Higher expression of G9a is correlated better with long-term survival of lung adenocarcinoma, and G9a inhibition enhances generation of the tumor-propagating cell phenotype to further promote metastasis and tumor progression (Rowbotham et al., 2018). Furthermore, it is interesting to see that hypoxic induction of these KMTs can have a number of phenotypic outcomes; either promoting or inhibiting cancerous phenotypes via Pontin or Reptin methylation, respectively (Lee et al., 2010, 2011). Thus, the role of G9a in regulating cancerous phenotypes also likely relates to the level of substrate protein. Therefore, we echo this caution in the use of pharmacological G9a inhibitors (Seton-Rogers, 2019). At least when considering the use of therapeutic strategies that rely on inhibition of G9a and/or GLP activity, it is crucial to consider the co-expression of substrate proteins as a deficiency in methylation may enhance tumorigenic potentials. To identify patients which may benefit from pharmacological G9a inhibition, Casciello and colleagues developed a G9a-suppressed gene signature associated with poor patient survival (Casciello et al., 2017). This methodology may act as a gauge for overall G9a activity and is based on epigenetic regulation. It would be interesting if this type of signature and decision may be based on the expression of modified non-histone substrates in the future.

## Conclusion and Future Perspective

Given the newly found hypoxia-inducibility of G9a and GLP methyltransferases, and the relevance of their corresponding substrates to hypoxia response pathways, we posit that hypoxia-inducible lysine methylation is a widespread event that directly influences the hypoxic PTM landscape. Indeed, at least two non-histone methylation events catalyzed by these enzymes have already been demonstrated to occur in an oxygen tension-dependent manner, influencing gene expression programs controlled by the HIF1 signaling pathway. Moreover, the hypoxia-inducibility of these enzymes appears to be contextual to cell type, therefore we anticipate that many of the methylation events described may be hypoxia-inducible solely in specific cells. Additionally, the potential hypoxia-inducibility of G9a and GLP non-histone methylation events has critical implications when considering function in the hypoxic tumor microenvironment. Lastly, the enzyme-substrate network of G9a and GLP methyltransferases is likely larger and more complicated than what has currently been studied. Thus, we anticipate the hypoxia-inducible nature of these methyltransferases to extend toward modulating the functions of other proteins within a plethora of biological contexts.

Overall, the mechanism dictating how oxygen acts as a signal for suppression of HIF is intimately involved in both human physiology and disease. The extent to which KMTs behave as non-canonical hypoxia-sensors, and the significance of this in the fundamentals of physiology and disease, are unknown at this time but we anticipate ongoing research to continue to unravel this possibility. Besides the writers of lysine methylation, research from our group and others relating to oxygen signaling through KDMs is continually demonstrating this PTM to be of relevance in this area. These perspectives are important when considering the widespread involvement of oxygen as a signaling molecule and we anticipate lysine methylation to be significantly more involved in the biochemical mechanisms underlying how cells sense and adapt to changes in oxygen supply.

## Author Contributions

AC, KB, WW, and WC conceptualized the article. AC wrote the manuscript and generated figures with revisions from KB, WW, and WC. All authors contributed to the article and approved the submitted version.

## Conflict of Interest

The authors declare that the research was conducted in the absence of any commercial or financial relationships that could be construed as a potential conflict of interest.
